# Population genetic structure and evolutionary history of Bale monkeys (*Chlorocebus djamdjamensis*) in the southern Ethiopian Highlands

**DOI:** 10.1186/s12862-018-1217-y

**Published:** 2018-07-10

**Authors:** Addisu Mekonnen, Eli K. Rueness, Nils Chr. Stenseth, Peter J. Fashing, Afework Bekele, R. Adriana Hernandez-Aguilar, Rose Missbach, Tanja Haus, Dietmar Zinner, Christian Roos

**Affiliations:** 1 Centre for Ecological and Evolutionary Synthesis (CEES), Department of Biosciences, University of Oslo, P.O. Box 1066, Blindern, N-0316 Oslo, Norway; 2 Department of Zoological Sciences, Addis Ababa University, P.O. Box: 1176, Addis Ababa, Ethiopia; 3 Department of Anthropology and Environmental Studies Program, California State University Fullerton, Fullerton, CA 92834 USA; 4 Primate Genetics Laboratory, German Primate Center, Leibniz Institute for Primate Research, Göttingen, Germany; 5 Cognitive Ethology Laboratory, German Primate Center, Leibniz Institute for Primate Research, Göttingen, Germany; 6 Gene Bank of Primates, German Primate Center, Leibniz Institute for Primate Research, Göttingen, Germany

**Keywords:** Primates, Genetic diversity, Hybridization, Gene flow, Mitochondrial DNA, Habitat fragmentation

## Abstract

**Background:**

Species with a restricted geographic distribution, and highly specialized habitat and dietary requirements, are particularly vulnerable to extinction. The Bale monkey (*Chlorocebus djamdjamensis*) is a little-known arboreal, bamboo-specialist primate endemic to the southern Ethiopian Highlands. While most Bale monkeys inhabit montane forests dominated by bamboo, some occupy forest fragments where bamboo is much less abundant. We used mitochondrial DNA (mtDNA) sequences to analyse the genetic structure and evolutionary history of Bale monkeys covering the majority of their remaining distribution range. We analysed 119 faecal samples from their two main habitats, continuous forest (CF) and fragmented forests (FF), and sequenced 735 bp of the hypervariable region I (HVI) of the control region. We added 12 orthologous sequences from congeneric vervets (*C. pygerythrus*) and grivets (*C. aethiops*) as well as animals identified as hybrids, previously collected in southern Ethiopia.

**Results:**

We found strong genetic differentiation (with no shared mtDNA haplotypes) between Bale monkey populations from CF and FF. Phylogenetic analyses revealed two distinct and highly diverged clades: a Bale monkey clade containing only Bale monkeys from CF and a green monkey clade where Bale monkeys from FF cluster with grivets and vervets. Analyses of demographic history revealed that Bale monkey populations (CF and FF) have had stable population sizes over an extended period, but have all recently experienced population declines.

**Conclusions:**

The pronounced genetic structure and deep mtDNA divergence between Bale monkey populations inhabiting CF and FF are likely to be the results of hybridization and introgression of the FF population with parapatric *Chlorocebus* species, in contrast to the CF population, which was most likely not impacted by hybridization. Hybridization in the FF population was probably enhanced by an alteration of the bamboo forest habitat towards a more open woodland habitat, which enabled the parapatric *Chlorocebus* species to invade the Bale monkey's range and introgress the FF population. We therefore propose that the CF and FF Bale monkey populations should be managed as separate units when developing conservation strategies for this threatened species.

**Electronic supplementary material:**

The online version of this article (10.1186/s12862-018-1217-y) contains supplementary material, which is available to authorized users.

## Background

The distribution and diversity of species are shaped by a combination of historical and contemporary factors. Currently, many species are affected by accelerated habitat destruction caused by both climate change and anthropogenic activity, the result being fragmentation, population decline and loss of genetic diversity [1–8]. The effects of habitat alteration are particularly detrimental to species with small geographic ranges and specialized niche requirements [2, 5, 9–11]. One such species severely affected by habitat fragmentation is the Bale monkey (*Chlorocebus djamdjamensis*) [12, 13]. This arboreal primate is endemic to the southern Ethiopian Highlands [14–16] and by far the most range-restricted of all green monkeys (genus *Chlorocebus*) [17, 18]. The taxonomy of green monkeys is disputed, but we here follow Groves [19] and accept the division of the genus into six species. In addition to the Bale monkey, Ethiopia harbours two other native, but not endemic *Chlorocebus* species, the vervet (*C. pygerythrus*) and the grivet (*C. aethiops*) [16, 19–21]. These two species are widely distributed, semi-terrestrial ecological generalists, inhabiting a variety of habitats and consuming a diverse diet of plant resources, invertebrates and small vertebrates [16, 22–24]. The Bale monkey, on the other hand, inhabits montane bamboo forests [14–16] where it feeds primarily on the young leaves and shoots of highland bamboo (*Arundinaria alpina*) [25]. Despite differences in habitat and dietary requirements, interspecific gene flow with grivets and vervets has been suggested to occur in the contact zones that are found in the fragmented part of the Bale monkey's range [12, 26]. A phylogenetic study by Haus et al. [20] revealed incongruences between mtDNA lineages and phenotypes in African green monkeys and suggested the occurrence of introgression between Bale monkeys and grivets as well as between vervets and grivets in Ethiopia.

**Fig. 1:**
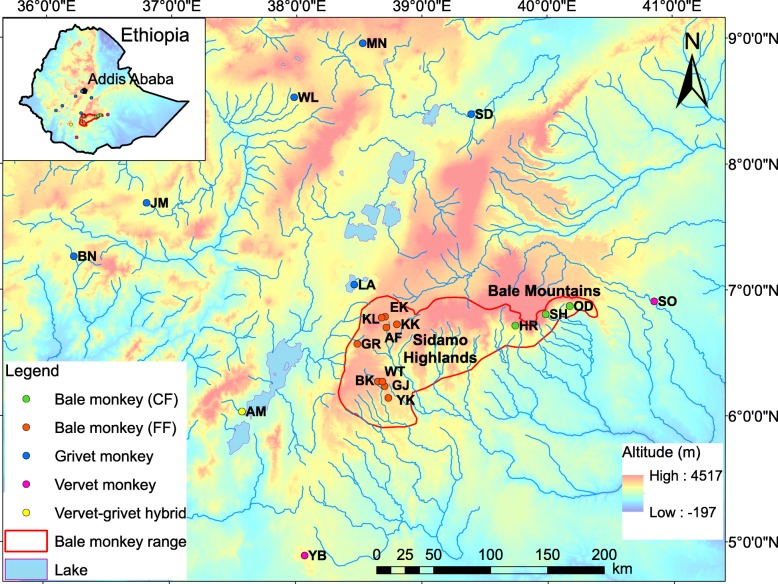
Map showing sampling localities for Bale monkeys, grivets, vervets and phenotypical vervet x grivet hybrids. The sampling sites in continuous forest (CF) covered more than 100 km^2^ of the Bale Mountains: Odobullu (OD), Shedem (SH), and Harenna (HR). The sampling sites in fragmented forests (FF) of the Sidamo Highlands are small and isolated habitats surrounded by human settlement, agriculture and/or grazing land: Kokosa (KK), Afursa (AF), Ekuma (EK), Kulla (KL), Gerbicho (GR), Bokata (BK), Wotiye (WT), Gejaba (GJ), and Yeko (YK). Grivet sampling sites: Lake Awassa (LA), Sodore (SD), Menagesha (MN), Woliso (WL), Jimma (JM), and Bonga (BN). Vervet sampling sites: Yabello (YB) and Sof Omar (SO). Vervet-grivet hybrid sampling site: Arba Minch (AM). The colour of localities corresponds to the clusters in Figs. 2 and 3

In modern times, conversion of the bamboo forest into agriculture and human settlement has resulted in population fragmentation in parts of the Bale monkey’s range. Although the species is locally abundant in the remaining continuous bamboo forests, e.g., Odobullu Forest [14, 15] (Fig. 1), populations found in forest fragments are generally small and declining and some have been extirpated in recent decades [12]. The total remaining population size of Bale monkeys is estimated to be less than 10,000 individuals [Mekonnen, unpublished data] with a declining trend [14, 15]. The species is classified as Vulnerable by the IUCN [15]. With the exception of the bamboo forests of Bale Mountains National Park (BMNP), most of the current Bale monkey range is located outside formally protected areas [14, 15] where the species is threatened by hunting and possibly by hybridization with grivets and vervets [12, 16, 20].

Bale monkeys are one of the least-studied African primates [25]. Therefore, baseline data on their phylogenetic position, population genetic structure and evolutionary history are crucial for assessing the conservation status of the taxon and for designing and implementing effective management strategies [7, 27–30]. Hence, we aimed to investigate the phylogeography and genetic diversity of Bale monkeys using the hypervariable region I (HVI) of the mitochondrial (mtDNA) control region (CR). This marker is rapidly evolving in vertebrates [31] and thus suitable to studying events that took place during the Pliocene-Pleistocene period. Particularly for female philopatric mammals, mtDNA markers have been considered more suitable than nuclear DNA markers to describe phylogeographic processes, even though they only reflect the maternal history [32, 33]. MtDNA has been widely used to detect molecular signatures of demographic changes [34–37]. However, a single-locus study will not adequately reflect the entire evolutionary history of a species [38, 39]. With so many of the world’s animal taxa currently threatened, single-locus studies are, nevertheless, useful when designing science-based management regimes aimed at enhancing the prospects of survival for little-studied species (e.g., [40]).

In particular, the main aims of the study were to investigate the following three objectives. First, we reconstructed phylogenetic relationships and estimated divergence times among Bale monkey populations and other green monkeys in Ethiopia. Second, we investigated genetic structuring within and between Bale monkey populations inhabiting continuous forest (CF) and fragmented forests (FFs). Third, we examined if any differences could be detected in the demographic histories of Bale monkey populations.

Our study leads to enhanced understanding on how rare specialist species may be affected by habitat alterations and demonstrates that genetic data, even from a single marker, can provide information that will be vital for future conservation efforts.

## Methods

### Study area and sample collection

The geographic range of the Bale monkey has been estimated as approximately 12,500 km^2^ [41]. Our study area covers the current species range in the southern Ethiopian Highlands, including both the CFs of the Bale Mountains [14] and the FFs of the Sidamo Highlands [12] (Fig. 1).

*Chlorocebus djamdjamensis*, *C. aethiops*, and *C. pygerythrus* were identified by phenotypic differences in their morphology, including coat colour, tail length and colour, facial hair (e.g., moustache) and whisker length [12, 16, 20]. Putative hybrids were identified by their intermediate coat colour, tail length, whisker length, and face colour and shape (Additional file 1). The presence of *C. aethiops* × *C. pygerythrus* hybrids with intermediate phenotypes at Arba Minch (Fig. 1) was described by Haus et al. [20].

Fresh faecal samples were noninvasively collected from May to December 2013 at three localities in CF and nine localities in FF (Fig. 1; Additional file 2). All of the FFs were small areas of less than 2 km^2^ embedded in a matrix of cultivated land and human settlements. The distance between the FFs varied between 3.5 km and 71.3 km through areas consisting of human settlements, grazing land, and cultivated land. Detailed information about sampling sites and samples are presented in Additional file 2.

Care was taken to avoid repeated sampling from the same individuals. In the FF habitat, where visibility was high, droppings were only collected from recognized individuals. In contrast, the CF habitat has areas with thick understory cover, where it was not always possible to sample only from identified individuals. Hence, we followed a particular group for up to one day and collected droppings within a short time interval whenever there was an opportunity [42, 43]. Since we collected only a few samples from each group – much less than the actual group sizes [13]– the probability of sampling any one individual more than once was small. We cut the surface of each dropping and transferred it into a 50 ml plastic tube containing silica beads for preservation. In some cases, the faecal samples were dried under a tree (to avoid direct sunlight that can cause DNA degradation) to remove excess moisture before preservation. We also replaced the silica beads if additional desiccation was required. To avoid contamination, clean disposable gloves were used when handling samples during drying and exchange of silica beads. For each sampling event, geographic coordinates (using Garmin GPSMap 62s), habitat type and group size were recorded. Samples were transported to Addis Ababa University and stored at − 20 °C until they were shipped to the University of Oslo, Norway for DNA extraction and further processing.

We also included 12 faecal samples of grivets (8), vervets (2) and putative grivet x vervet hybrids (2) previously collected by Haus et al. [20] from nine localities in southern Ethiopia (Fig. 1; Additional file 2). These samples were kept for at least 24 h in ethanol (> 90%) and then stored on silica beads after drying [20]. Further details about the collection of these samples are outlined in Haus et al. [20].

### Ethical statement

Permission to conduct this research was granted by the Ethiopian Wildlife Conservation Authority in compliance with the Convention on International Trade in Endangered Species of Wild Fauna and Flora (CITES). Faecal samples were collected non-invasively without harming or disturbing the animals. This study meets all animal care policies and adheres to the legal requirements of Ethiopia, Norway, and Germany. It also complied with the ethical and legal requirements of the American Society of Primatologists Principles for the Ethical Treatment of Primates.

### Laboratory work

We extracted DNA from faecal samples following the protocol described in Atickem et al. [44]. A thin slice (≤ 0.01 g) was cut from the surface of each sample using a clean razor blade and transferred to an Eppendorf tube containing 300 μl lysis buffer (500 mM Tris, 10 nM NaCl, 50 mM EDTA). After 30 min incubation at 56 °C and 1 min centrifugation, 100 μl of the lysate was transferred to a tube containing 95 μl isopropanol for DNA precipitation and 20 μl of Dynabeads® MyOne™ SILANE (Invitrogen Dynal AS Oslo Norway) for DNA binding. The tubes were then left for 2 min on a magnetic device before the supernatant was discarded. The DNA was washed twice with 200 μl 70% ethanol and finally eluted in 100 μl mqH_2_O preheated to 80 °C. We included one negative control per eight sample extractions.

DNA extraction from faecal samples collected by Haus et al. [20] was conducted with the QIAamp DNA Stool Mini Kit (Qiagen, Hilden, Germany) following standard protocols with only minor modifications [20]. All DNA extracts were stored in 50 μl aliquots at − 20 °C until further processing. DNA concentrations were measured on a NanoDrop ND-1000 spectrophotometer (Thermo Fisher Scientific, Waltham, USA) or Qubit 2.0 (Thermo Fisher Scientific).

We amplified an approximately 800 bp long fragment of the HVI region from 131 samples. We conducted PCR reactions in a total volume of 30 μl containing a final concentration of 0.33 μM of each of the genus-specific primers 5’-AAATGAACTTGCCCTTGTAG-3′ and 5’-GGTGTTGCGTGCAGACC-3′, 3 mM MgCl_2_, 0166 mM dNTPs, 1× buffer, 1 U Biotherm Taq DNA polymerase (Genecraft, Cologne, Germany) and 100 ng DNA. The cycling conditions consisted of a pre-denaturation step at 94 °C for 2 min, followed by 40–50 cycles, each with denaturation at 94 **°**C for 1 min, annealing at 54 **°**C for 1 min, and extension at 72 **°**C for 1 min. At the end, a final extension step at 72 **°**C for 5 min was added. We checked PCR performance on 1% agarose gels. PCR products were excised from the gel, cleaned with the QIAquick Gel Extraction Kit (Qiagen, Hilden, Germany) and Sanger-sequenced in both directions on an ABI 3130*xl* DNA sequencer (Applied Biosystems, Foster City, USA) using the BigDye Cycle Sequencing Kit and the amplification primers. Sequence electropherograms were checked by eye with 4Peaks 1.8 (www.nucleobytes.com) and sequences were assembled and manually edited in SeaView 4.4.0 [45]. Sequences were deposited in GenBank and are available under the accession numbers MG786940 - MG787070.

To avoid cross-sample contamination in the laboratory, all working steps (DNA extraction, PCR setup, PCR amplification, gel electrophoresis, PCR product purification, and sequencing) were conducted in separate and therefore dedicated laboratories under Captair Bio PCR cabinets (Erlab, Val de Reuil, France). Benches were cleaned with 10% bleach and gloves were regularly changed. Further, PCR controls (without template DNA) were routinely conducted and procedures were repeated for 10% of randomly selected samples. To minimize the risk of amplifying nuclear mitochondrial-like sequences (numts), we designed genus-specific primers on the basis of published mtDNA genomes from *Chlorocebus* [46]. We tested these primers, using the lab methods mentioned above, in ten *Chlorocebus* individuals for which we recently generated mtDNA genomes [46]. The obtained sequences were identical to their mtDNA genome orthologs, suggesting that the primers amplify solely mtDNA and no numts.

### Data analyses

#### Phylogenetic reconstruction and divergence time estimation

For phylogenetic tree reconstructions, we added an orthologous sequence from *Chlorocebus sabaeus* (EF597503.1) to our dataset as an outgroup. Sequences were aligned with MUSCLE 3.8.31 [47], implemented in MEGA 7.0.14 [48] and inspected by eye in BIOEDIT 7.2.5 [49]. The best-fit nucleotide substitution model (HKY) [50] was selected using the Bayesian Information Criterion (BIC) [51] as implemented in jModeltest 2.1.6 [52]. We constructed phylogenetic trees using both maximum-likelihood (ML) and Bayesian methods. A ML tree was constructed in MEGA with the nearest Neighbor-Interchange by bootstrapping 10,000 replicates. To reconstruct a Bayesian phylogenetic tree and to estimate divergence times, we applied the BEAST package 2.4.4 [53, 54]. Since no reliable fossil-based calibration points are available, divergence ages were calibrated based on the mtDNA split between *C. sabaeus* and all other *Chlorocebus* spp. using a normal distribution with a mean of 3.50 Mya and a 95% highest posterior density (HPD) of 3.10–3.90 Mya [46]. We implemented the HKY model of nucleotide substitution with a relaxed uncorrelated lognormal clock model and a Yule model as tree priors. We conducted two Markov Chain Monte Carlo (MCMC) runs, each with 10 million generations, with trees sampled every 10,000 generations. Tracer 1.6 was used to investigate performance with a 10% burn-in and to verify that the effective sample size (ESS) was greater than 200. LogCombiner 2.4.4 was used to combine independent runs and TreeAnnotator 2.4.4 was applied to generate a consensus tree using maximum clade credibility with median node heights. We visualized and summarized the tree using the FigTree 1.4.2 drawing tool. We defined all clades with both bootstrap (BS) and posterior probability (PP) support of > 90% and > 0.90, respectively as significantly monophyletic.

To further trace phylogenetic relationships among haplotypes from all Bale monkeys and 12 other green monkeys, we constructed a TCS network (based on the method of Templeton et al. [55] that is particularly suitable to infering population level genealogies [56]) using the software PopART 1.7 [57].

### Genetic diversity and population genetic structure

We estimated genetic diversity for each Bale monkey locality, CF, FF and overall populations as the number of haplotypes (a unique base sequence found in one or more individuals), haplotype diversity, nucleotide diversity and number of polymorphic sites [58] using Arlequin 3.5.2.2 [59]. When the sampling sites were merged into CF and FF, the number of individuals was *n* = 34 and *n* = 85, respectively. For the purpose of comparison, we also included 12 sequences from other green monkeys.

We calculated genetic differentiation among local Bale monkey populations as pairwise fixation indices (F_ST_) in Arlequin. We ran 10,000 permutations to assess if the population pairs were significantly (0.05% significance level) more differentiated than what would have been expected if haplotypes were randomly distributed among them. Theoretically, F_ST_ values range from 0 (no genetic differentiation) to 1 (complete genetic differentiation). We applied a Mantel’s test [60] to assess if the correlation between pairwise genetic (F_ST_) and geographic distances (km) among all sampling sites were higher than what would be expected for a randomly reproducing population. The geographic distances were estimated using Geospatial Modeling Environment and ArcGIS 10.3 following Mekonnen et al. [13] and the Mantle’s test was performed in IBDWS 3.15 [61] with 1000 permutations and a 95% confidence interval (CI). We analysed population genetic structure and differentiation within and between Bale monkey populations using Analysis of Molecular Variance (AMOVA) as implemented in Arlequin. Variance components within and among populations were calculated with 10,000 random permutations. In addition, we calculated pairwise genetic distances between populations and/or taxa using a Kimura-two-parameter (K2P) model as implemented in MEGA with 10,000 replicates.

### Population demographic history

We tested for molecular signatures of demographic changes (sudden fluctuations in population size) in the evolutionary history of the Bale monkeys (CF, FF and overall) by running three widely used tests (e.g., [34, 62, 63]). First, we applied neutrality tests using Fu’s *F*_S_ [64] and Tajima’s *D* [65] in Arlequin with 10,000 permutations. Second, we examined mismatch distribution of pairwise differences between sequences as implemented in Arlequin and DnaSP 5.10.1 [66] with 10,000 bootstrap replicates. The statistical significance was determined by testing the goodness-of-fit between the observed and expected mismatch distributions, using the raggedness index (*r*) [67] and the sum of squared differences (SSD) before (θ_o_) and after expansion (θ_1_) [68, 69]. Studies have demonstrated that the shape of the mismatch distribution generally exhibits multimodal and ragged distributions for stationary and non-expanding populations, whereas unimodal or smooth distributions indicate that populations have experienced historical demographic expansions or bottlenecks [68, 70]. Third, the demographic history of Bale monkey populations was inferred to assess effective population size changes using the Bayesian Skyline Plot (BSP) method [71] as implemented in BEAST. The BSP model assumes a single panmictic population and violation of this assumption can result in misleading demographic inferences [72, 73]. Although BSP assumes a single species or monophyletic group in analyses of demographic history, similar analyses have been carried out when modelling hybridization at population peripheries [74, 75]. Thus, we generated BSPs for genetically homogenous geographic populations as inferred by AMOVA (e.g., [34, 74, 75]). As substitution models, we applied HKY for the CF population and HKY + I for the FF population as they were chosen as best-fit models by jModeltest. The analyses were carried out using a relaxed uncorrelated lognormal clock with a coalescent Bayesian Skyline priori and a random starting tree. Time to the most recent common ancestor (MRCA) for each population was set to analyse their corresponding BSP using normal distribution. Two independent analyses were run for a total of 30 million MCMC generations sampling every 3000 generations with 10% of the samples as burn-in. The results of each run were checked to ensure convergence and stationarity using Tracer. Runs, where ESS values were less than 200 for all parameters, were discarded.

## Results

### Phylogeny and estimation of divergence time

The final alignment had a length of 735 bp and contained 132 sequences, which were derived from samples of 119 Bale monkeys, eight grivets, two vervets, two individuals identified as grivet x vervet hybrids and one *C. sabaeus* sequence as an outgroup. Numts are highly unlikely to be present in our dataset, as we (1) used only faecal material in which nuclear DNA is largely degraded [76], (2) the HVI region was amplified with genus- and mtDNA-specific primers, and (3) no multiple peaks were obtained by direct sequencing of PCR products. We found 201 polymorphic sites, of which 168 were parsimony informative, and 33 were singletons. The alignment comprised of 26 haplotypes, of which 16 (H1-H16) were derived from phenotypical Bale monkeys, six (H17-H22) from phenotypical grivets, two (H23-H24) from phenotypical vervets, and two (H25-H26) from phenotypical grivet x vervet hybrids (Additional file 3).

**Fig. 2:**
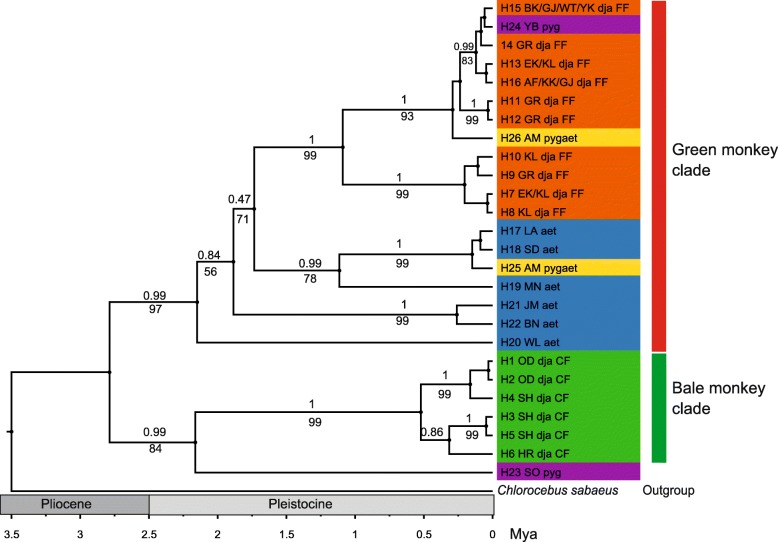
Ultrametric tree showing phylogenetic relationships and divergence ages among mtDNA haplotypes (H1-H26). Numbers above and below branches represent Bayesian (PP) and ML (BS) support values, respectively. Only clades with node support of PP > 0.90 and BP > 90% are considered monophyletic and scaled as million years before present (Mya). For haplotype names, species affiliations (based on phenotype), locality codes and forest types (CF and FF) see Fig. 1; Additional file 2. Colours represent groups: green - Bale monkey in CF; orange - Bale monkey in FF; blue - grivet; purple - vervet; yellow - vervet-grivet hybrid

The topology of our phylogenetic tree is similar to that of Haus et al. [20] and suggests two major clades for Ethiopian green monkeys (Fig. 2). One clade comprises all Bale monkey haplotypes from CF and represents a sister lineage to a vervet haplotype (H23) from Sof Omar, whereas the second clade contains all haplotypes from FF Bale monkeys as well as from vervets, grivets and their putative hybrids. Within the first clade, the CF Bale monkeys form a monophyletic group, which corresponds to clade C5 of Haus et al. [20], whereas in the second clade the FF Bale monkeys form a subclade which also contains haplotypes of *C. pygerythrus* and of *pygerythrus*/*aethiops* hybrids and corresponds to clade C2 of Haus et al. [20]. The FF Bale monkey subclade forms a sister clade to several *C. aethiops* and *pygerythrus*/*aethiops* hybrid lineages. The vervet haplotype (H23) from Sof Omar represents clade C6 of Haus et al. [20]. We will hereafter refer to the CF clades as Bale monkey clade and the FF clade as green monkey clade.

We estimated the divergence ages between the green monkey and Bale monkey clades at 2.79 (2.21–3.71) Mya, and the split of the Bale monkey clade from its sister lineage, H23 from Sof Omar, at 2.16 (1.41–2.3.21) Mya (Fig. 2). These divergence ages are similar to those based on complete mtDNA genomes by Dolotovskaya et al. [46]. The age of the MRCA of the clade containing all of the haplotypes from FF Bale monkeys was estimated at 1.09 (0.61–1.81) Mya and that of the Bale monkey clade at 0.53 (0.25–0.93) Mya.

**Fig. 3:**
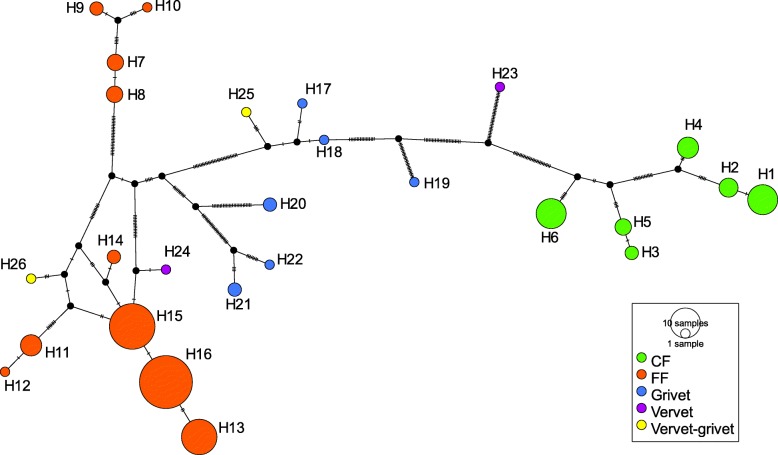
A TCS network of mtDNA haplotypes from Ethiopian *Chlorocebus* taxa. Coloured circles represent individual haplotypes and the sizes of the circles are proportional to the frequency of the haplotypes. Each point mutation is symbolized by a mark on the lines connecting haplotypes. The colours are used to visualize the following groups: green - Bale monkey in CF; orange - Bale monkey in FF; blue - grivet; purple - vervet and yellow - vervet-grivet hybrids. Small black dots indicate missing intermediate haplotypes

The two distinct clusters of CF and FF Bale monkey haplotypes also appeared in our TCS network (Fig. 3), although the pattern is more obscure here due to the location of several grivet, vervet and hybrid haplotypes between and within the two Bale monkey clusters.

### Genetic diversity and population differentiation

Among the 16 Bale monkey haplotypes identified, only four were shared by two or more localities within the FF population, while the remaining 12 (75%) haplotypes were found only at one locality (Table 1; Additional file 3). H16 was the most frequent haplotype found in 31 of the Bale monkey samples (26.1%), while H10 and H12 were the rarest and found only in one individual each (Table 1). The genetic diversity parameters in terms of number of haplotypes (h), haplotype diversity (Hd), nucleotide diversity (π) and number of polymorphic sites (p) for each locality, the CF, FF and the overall Bale monkey populations are presented in Table 1. The highest haplotype diversity (0.778) was calculated for Kulla (FF) with four haplotypes, followed by Gerbicho (FF) and Shedem (CF). We observed similar levels of haplotype diversity for the CF (0.804) and FF (0.768) Bale monkey populations. Five of the nine FF localities and one of the three CF localities exhibited only one haplotype (Table 1). Overall, we observed higher nucleotide diversity in CF (0.0359) than in FF (0.0156).

**Table 1:** Genetic diversity indices for Bale monkeys and grivets

Genetic diversity within sampling localities											
Sample localities (Codes)	Forest type	n	h	h/n	Uh	Sh	NUh	NSh	p	Hd ± SD	π ± SD
Odobullu (OD)	CF	14	2	0.14	2	0	H1, H2	0	1	0.440 ± .0.112	0.0006 ± 0.0001
Shedem (SH)	CF	10	3	0.30	3	0	H3, H4, H5	0	41	0.689 ± 0.104	0.0309 ± 0.0168
Harenna (HR)	CF	12	1	0.10	1	0	H6	0	0	0	0
Kokosa (KK)	FF	14	1	0.07	0	1	0	H16	0	0	0
Afursa (AF)	FF	11	1	0.09	0	1	0	H16	0	0	0
Ekuma (EK)	FF	11	2	0.27	1	2	0	H7, H13	46	0.182 ± 0.014	0.0124 ± 0.0070
Kulla (KL)	FF	10	4	0.40	2	2	H8, H10	H7, H13	51	0.778 ± 0.091	0.0378 ± 0.0205
Bokata (BK)	FF	6	1	0.17	0	1	0	H15	0	0	0
Wotiye (WT)	FF	3	1	0.33	0	1	0	H15	0	0	0
Gejaba (GJ)	FF	10	2	0.20	0	2	0	H15, H16	1	0.533 ± 0.095	0.0008 ± 0.0001
Yeko (YK)	FF	10	1	0.10	0	1	0	H15	0	0	0
Gerbicho (GR)	FF	10	4	0.40	4	0	H9, H11, H12, H14	0	52	0.733 ± 0.120	0.0293 ± 0.0160
Genetic diversity within two populations (CF and FF)											
CF population	CF	34	6	0.18	6	0	H1-H6	0	57	0.804 ± 0.035	0.0359 ± 0.0179
FF population	FF	85	10	0.12	10	0	H7-H16	0	60	0.768 ± 0.028	0.0156 ± 0.0079
Overall Bale monkeys		119	16	0.13	12	4	H1-H6, H8-H12, H14	H7, H13, H15, H16	192	0.867 ± 0.017	0.0845 ± 0.041
Overall genetic diversity from all six grivet localities (LA, SD, MN, WL, JM and BN)											
Overall grivets		8	6	0.75	6	0	H17-H22	0	117	0.929 ± 0.084	0.0762 ± 0.04216

Genetic diversity as measured for each sampling locality and the CF and FF populations as well as for grivets (localities combined). *n* = number of individuals sampled, *h* = number of haplotypes, *h/n* = adjusted number of haplotypes, *Uh* = number of unique haplotypes, *Sh* = number of shared haplotypes, *NUh* = name of unique haplotypes, *NSh* = name of shared haplotypes, *p* = number of polymorphic sites, *Hd* = haplotype diversity, and π = nucleotide diversity

We found significant genetic differentiation (pairwise F_ST_) among most sampling localities of Bale monkeys, except between a few of the FF sites (*p* > 0.05, Additional file 4). The high F_ST_ values observed between some population pairs are explained by the absence of shared haplotypes. The AMOVA results suggested that as much as 87% of the total variability was explained by differentiation between the CF and FF populations (Table 2). The differentiation among sampling localities within populations explained 7.9%, and variability within locality explained 5.1% of the variation (Table 2). Further, we found a significant correlation between genetic and geographic distances (km) among all sampling localities of Bale monkeys (Mantel *r* = 0.66; *p* = 0.002), suggesting that the genetic structure of the Bale monkey follows a pattern of IBD. When testing for IBD among the FF sampling sites alone, the result was not significant (Mantel *r* = 0.342; *p* = 0.06). The genetic distance between CF and FF populations was estimated to be 14.1%, which is higher than the distance between CF and grivets (13.4%) and FF and grivets (9.8%).

**Table 2:** Comparison of geographical structure between CF and FF Bale monkey populations by AMOVA

Comparison	df	SSD	% of variation	Fixation indices	*P*-value
Between populations	1	2775.17	87.01	F_CT_: 0.8701	0.0049
Among sampling localities within populations	10	522.67	7.86	F_SC_: 0.6052	0.0000
Within sampling localities	107	352.58	5.13	F_ST_: 0.9487	0.0000

### Demographic history of Bale monkeys

**Fig. 4:**
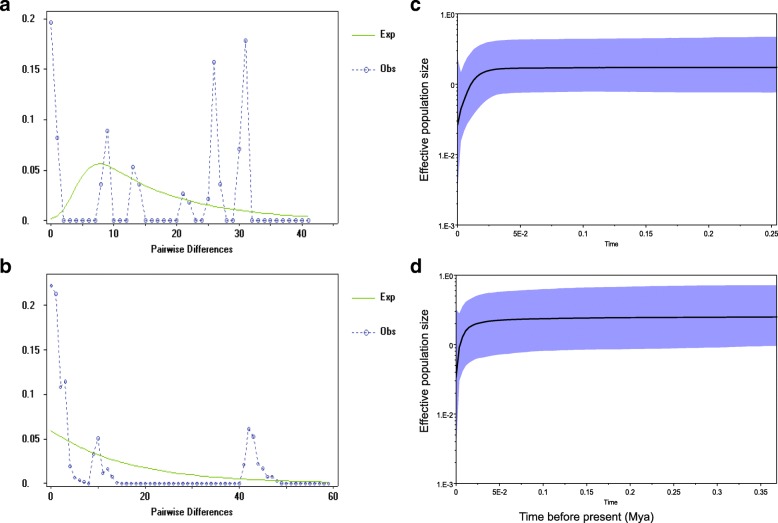
Mismatch distributions (left) and Bayesian skyline plots (right) of Bale monkey populations (CF and FF). Mismatch distributions for (**a**) CF population and (**b**) FF population. Dotted lines indicate observed frequencies of pairwise haplotype differences and solid lines denote the expected frequencies under a model of population expansion. Bayesian skyline plots for CF population (**c**) and FF population (**d**). The x-axis represents time in million years ago (Mya) and the y-axis shows effective population size of females (N_e_) multiplied by generation time (T) in a log scale. Black lines denote the median effective population size (*N*_*e*_*T*) over time to the present and blue shaded areas represent the 95% HPD limits

The demographic history was analysed for the overall Bale monkey population as well as for the CF and FF populations separately. The CF population showed non-significant positive values for Fu’s *F*_S_ and Tajima’s *D* (Table 3) indicating a stable demographic history. This result was corroborated by the highly ragged and multimodal mismatch distribution patterns with sharp peaks (Fig. 4a). The FF population showed non-significant positive values for Fu’s *F*_S_ suggesting a stable demographic history. However, a small negative value for Tajima’s *D* suggested population expansion*,* though this result was not statistically significant. The mismatch distribution observed for the FF population was nearly multimodal, suggesting that the population has not undergone recent population expansion (Fig. 4b). Finally, the overall Bale monkey population showed non-significant positive values for both Fu’s *F*_S_ and Tajima’s *D* (Table 3), again characteristic of a stable demographic history with stable population size. In addition, the mismatch distribution model showed multimodal and moderately ragged distribution patterns, suggesting demographic stability. In sum, the mismatch and neutrality analyses suggested that the CF, FF and overall Bale monkey populations have had stable population sizes and have not undergone recent population expansions. Likewise, BSPs revealed that the CF and FF Bale monkey populations generally showed prolonged demographic stability before they started to decline around 25,000 years ago (Figs. 4c and d).

**Table 3:** Summary of demographic history of Bale monkey populations

	CF population	FF population	Overall population
Sample size	34	85	119
π	26.210	10.542	61.871
rg (*p*-value)	0.1204 (0.000)	0.02449 (0.942)	0.0167 (0.7393)
FU’s Fs (*p*-value)	23.388 (1.000)	11.187 (0.987)	34 × 10^37^ (1.000)
Tajima’s D (*p*-value)	2.810 (0.999)	−0.393 (0.412)	1.910 (0.976)
SSD (*p*-value)	0.0933 (0.000)	0.0221(0.877)	0.0354 (0.526)

Average number of pairwise differences (π); neutrality tests of Raggedness index (rg), Fu’s F test and Tajima’s D test, and the sum of squared deviation (SSD)

## Discussion

Our genetic analyses (TCS network, phylogenetic trees and AMOVA) suggest a division of Bale monkeys into two distinct clusters corresponding to the geographic structuring between CF (Bale Mountains) and FF (Sidamo Highlands). No mtDNA haplotypes are shared between these clusters (Fig. 1). Notably, the haplotypes of the FF Bale monkeys clustered with grivet and vervet haplotypes rather than with those from their conspecifics in CF.

### Phylogeny and divergence time

In general, our phylogeny and divergence ages are consistent with those based on complete cytochrome b sequences [20] or complete mtDNA genomes [46]. Similar to previous studies, we found a lack of taxonomic clustering among Ethiopian *Chlorocebus* monkeys, strengthening the notion that mtDNA sequence analyses may not be suitable for taxonomic delimitation in *Chlorocebus*. In contrast to previous studies, which included just one specimen per taxon, our results demonstrate that the poly- and paraphyletic relationships remain among Ethiopian *Chlorocebus* monkeys even when a larger number of specimens are included. Interestingly, our results show that Bale monkeys living in their original undisturbed habitat (CF) form a monophyletic group suggesting no indication of hybridization, whereas the population in the fragmented and disturbed habitat (FF) clustered with vervets and grivets suggesting the FF population most likely represents an introgressed deme.

Hybridization across species boundaries is not uncommon when closely related taxa meet in contact zones [26, 74, 77, 78]. Hybridization is also not unusual between *Chlorocebus* species. Indeed, it was already suggested to occur by Haus et al. [20] and recently confirmed in a whole genome study [79]. However, no Bale monkeys were included in the latter study. Nevertheless, observations on phenotypes suggest interspecific gene flow in some localities of FF Bale monkeys. In particular, putative phenotypic Bale monkey × grivet hybrids were recorded [12] and one Bale monkey × vervet hybrid was observed at Kokosa with intermediate coat colour, tail length, and whisker length [J.-M. Lernould, personal communication, A. Mekonnen, personal observation] (Additional file 1). The majority of phenotypes observed in FF suggest that this population is most likely a relict *C. djamdjamensis* population, which was introgressed by vervet and grivet monkeys. The FF populations may have captured their mtDNA haplotypes from grivets and vervets via female-mediated gene flow (e.g., [80, 81]), while retaining most of the phenotypic features and ecological behaviours (e.g., bamboo niche – when available – folivory and arboreality) of the typical Bale monkey [82–84]. However, slight differences between the CF and FF Bale monkeys in morphology [12] (Additional file 1), behavioural ecology [13, 83, 84] and gut microbiota [82] further support the hypothesis that gene flow has already altered the gene pool of the FF population, making these monkeys ecologically flexible and more similar to other *Chlorocebus* species [13, 83, 84]. Here, whole genome analyses will certainly help to solve the question of adaptation by hybridization.

We did not detect any haplotypes shared between CF and FF Bale monkey populations (Figs. 2 and 3) indicating that gene flow between the demes is not common. The CF and FF sampling sites are separated by a distance of about 100 km. Although sampling for the current study provides very good coverage of the distribution range, some Bale monkey sites are known to exist in the area between the CF and FF sites sampled. It is thus possible that intermediate haplotypes can be found here and future sampling from this area would contribute to an improved view of historic and contemporary gene flow in these primates.

If the FF deme is indeed an introgressed relict population of Bale monkeys, it is reasonable to ask why hybridization has occurred here but not in the CF deme? A possible explanation could be that the habitat in the western part of the Bale monkey’s range, i.e. in FF, changed from the original bamboo dominated forest into a more open woodland and fragmented habitat [12, 15, 16], thus making it more suitable for the generalist vervet and grivet monkeys. Whether the habitat change was a consequence of natural climate change or the result of anthropogenic habitat alteration cannot be resolved at present. However, we suspect that both factors played a role and that human impact has probably been most influential over the last few centuries.

### Population genetic structure

Although Bale monkeys have been extirpated in some FF localities in recent decades [12] and several FF localities lack genetic diversity, overall we found nearly similar levels of haplotype diversity in CF and FF populations (Table 1). However, the pattern of how haplotypes were distributed among sampling sites differed remarkably within the CF and FF populations (Fig. 3; Table 1). Within CF, none of the haplotypes were shared among sites, whereas eight out of nine FF sites (except Gerbicho) shared one or more haplotypes with other FF sites (Table 1). When several haplotypes were found within a FF sampling site, they were not necessarily closely related (e.g., H7 and H13, Table 1; Fig. 3), a pattern which is suggestive of genetic drift (random changes in haplotype frequency from generation to generation) [85]. The haploid nature of mtDNA and the fact that it is only maternally inherited leads to an effective population size that is four times smaller compared to autosomal nuclear DNA. The influences of genetic drift and population bottlenecks will thus be more clearly expressed, with large inter-population differentiation, when working with mtDNA [86]. Like many cercopithecines, including the other *Chlorocebus* spp. [23, 87], Bale monkeys are believed to exhibit male dispersal and female philopatry. It is thus possible that nuclear markers would display less differentiation among sampling sites due to male gene flow. Presently it is, however, unlikely that Bale monkey males move between isolated sites in the FF due to human disturbance [12, 15]. A number of studies have shown that habitat fragmentation may affect genetic structure by limiting movement between demes, and hence gene flow [10, 11, 88, 89]. The Bale monkey’s arboreal lifestyle and specialized niche [25, 84] limit its dispersal ability and thus make it particularly prone to genetic isolation due to extensive gaps in suitable habitat. The habitat gaps may be caused by human disturbance, climate change, and the landscape features of the southern Ethiopian Highlands (e.g., deep gorges and alpine areas above the tree line) [90].

### Demographic history

Like for other African green monkeys [91], we found no genetic evidence for historical population expansion for the Bale monkey populations (Table 3). The recent population decline, as revealed by BSP, might be explained by climate change during and following the Last Glacial Maximum (LGM, 23,000–18,000 years before present), a period characterized by cool and wet climatic conditions in the southern Ethiopian Highlands [92]. Since the Bale monkey populations are confined to a narrow geographic range with restricted suitable habitat (bamboo forest) [14], a reduction of suitable habitat and thus a reduction of the species’ range is to be expected. Currently, the FF populations are restricted to small isolated forest fragments mainly resulting from anthropogenic habitat modification [12, 15], which probably had an additional negative impact on Bale monkey population size in the last few centuries. We do note, however, that our BSP-based inferences into the demographic history of Bale monkeys should be interpreted with caution because of the confounding effect of the strong population structure that can lead to false inferences of population decline [72, 93, 94]. Further, we used a single mtDNA locus which is not likely to reflect the complete demographic history of the species (cf., [95]).

### Implications for conservation

Bale monkeys are currently at high risk of extinction because of habitat alteration, hunting and possibly hybridization [12, 15, 41]. Science-based management strategies may thus be the only means to ensure the species’ long-term persistence. Studies of population genetic structuring of mtDNA have been applied to help identify management units appropriate for the conservation of endangered species [27–29, 40, 96]. The results of our study suggest two isolated Bale monkey populations, of which one (FF) most likely consists of hybrids with other *Chlorocebus* species. We therefore propose that two separate management units should be defined when designing strategies for the long-term conservation of Bale monkeys to preserve their unique genetic diversity and evolutionary potential.

Because the CF population represents what is believed to be the typical Bale monkey population – and is not sympatric with any other *Chlorocebus* species – we propose that this population warrants special conservation attention. We therefore recommend improved protection of continuous bamboo forest habitats by minimizing logging of bamboo for local consumption and commercial purposes. The FF population of the Sidamo Highlands is rapidly shrinking due to increasing anthropogenic impacts [12, 15, 41]. We therefore suggest connecting forest fragments to increase gene flow between isolated populations and prevent loss of genetic diversity, thereby promoting the long-term survival of these populations [97, 98]. Further, hybridization/introgression between Bale monkeys of the FF populations and the widely distributed vervets and grivets may have consequences for conservation. Provisionally, the FF population should be managed separately from parapatric vervets and grivets, at least until hybridization among them is confirmed. The impacts of hybridization on the conservation strategies for rare and threatened taxa can be complex and controversial [99, 100]. Hybridization may help to rescue small populations through increasing genetic variation by replacing parental genes with adaptive hybrid genes and consequently increasing the potential for adaptation in a changing environment and sometimes the formation of new species or subspecies [74, 101–104]. On the other hand, hybridization may lead to the extinction of rare and endangered species through genetic swamping of native populations [105–107]. When hybridization occurs in a large geographic range, it can cause a decrease in the total population size of native and range-restricted species through the loss of historically original populations. Conversely, hybridization can result in range expansion for non-endangered and widely adapted species [108]. Such events could negatively impact the total population size and conservation status of the rare and specialist Bale monkey given that is surrounded by two widely distributed, generalist sister species in southern Ethiopia.

## Conclusions

In our study, we demonstrated strong genetic differentiation between Bale monkeys from the Bale Mountains (CF) and the Sidamo Highlands (FF). Populations from the two habitat types differ not only in mtDNA but also in morphology [12] (Additional file 1), ecology and behaviour [13, 83, 84] as well as in gut microbiota [82]. The differentiation was most likely initiated by habitat alteration due to past climate change. Bamboo forests, suitable for Bale monkeys, were replaced by a more open woodland habitat in the western part of the species' range, making introgression by parapatric *Chlorocebus* species possible. This alteration of the bamboo forest was later intensified and accelerated by human activities. As a consequence of genetic differentiation, we propose that the CF and FF Bale monkey populations should be managed as separate units. Overall, the results of this study increase our general understanding of how habitat fragmentation, hybridization and geographic isolation together have shaped the genetic structure of a rare, range-restricted and specialist primate. Future research focusing on bi-parentally and paternally inherited genetic markers, as well as morphological and ecological variability within the species, will be needed to further increase our understanding of the evolutionary history of this unusual species.

## Additional files

Additional file 1: Comparison of the phenotypic appearance of typical Bale monkeys, vervets, and their putative hybrids. (a) Adult putative vervet at Kokosa possibly the father of the hybrid. (b) Adult female Bale monkey and a putative juvenile hybrid of Bale monkey (♀) × vervet (♂) at Kokosa. A putative hybrid at Kokosa (c, d) with an intermediate coat colour that is more golden than that of a Bale monkey but less golden than that of a vervet. The animal shows a white browband, though it is smaller than that of a vervet and nearly absent in Bale monkeys. The hybrid has an intermediate tail length and face colour, the face is darker in vervet but lighter in Bale monkey. However, this hybridization event is most likely as a consequence of the release of a pet vervet in the range of the FF population [J.-M. Lernould, personal communication]. (e, f) Adult vervet from Gonosa, Robe District with more golden fur, hands and feet are very dark, thick white browband, long and white whiskers, dark face with golden moustache, and long tail with black tip. (g, h) Adult Bale monkey in CF of Odobullu Forest exhibits a relatively brown/grey coat, lacks a white browband, whiskers are short, front neck covered with white fur, hands and feet blackish brown, and relatively short tail [12]. Bale monkey in FF of Kokosa (i) and Afura (j) that are phenotypically more similar to CF population (g, h) than that of grivet [12] and vervet (e, f). (DOCX 5055 kb)

Additional file 2: Summary of sampling localities and number of samples. (DOCX 24 kb)

Additional file 3: Summary of haplotype distribution of *Chlorocebus* species. (DOCX 23 kb)

Additional file 4: F_ST_ values (*p* values) of pairwise comparisons among 12 Bale monkey localities. (DOCX 26 kb)

## Abbreviations

AMOVA: Analysis of Molecular Variance
BIC: Bayesian Information Criterion
BS: Bootstrap
BSP: Bayesian Skyline Plot
CF: Continuous forest
CI: Confidence interval
CITES: Convention on International Trade in Endangered Species of Wild Fauna and Flora
CR: Control region
ESS: Effective sample size
FF: Fragmented forest
HVI: Hypervariable region I
IUCN: International Union for Conservation of Nature
LGM: Last Glacial Maximum
MCMC: Markov Chain Monte Carlo
ML: Maximum-likelihood
MRCA: Most recent common ancestor
mtDNA: mitochondrial DNA
Mya: Million years ago
PP: Posterior probability
SSD: Sum of squared differences
