# Supplementary material for: Population genetic structure and evolutionary history of Bale monkeys (Chlorocebus djamdjamensis) in the southern Ethiopian Highlands
Source: BMC Evol Biol. 2018 Jul 10;18:106. doi: 10.1186/s12862-018-1217-y (PMC6038355; doi:10.1186/s12862-018-1217-y)
Supplement: Supplementary file 1 — Comparison of the phenotypic appearance of typical Bale monkeys, vervets, and their putative hybrids. (a) Adult putative vervet at Kokosa possibly the father of the hybrid. (b) Adult female Bale monkey and a putative juvenile hybrid of Bale monkey (♀) × vervet (♂) at Kokosa. A putative hybrid at Kokosa (c, d) with an intermediate coat colour that is more golden than that of a Bale monkey but less golden than that of a vervet. The animal shows a white browband, though it is smaller than that of a vervet and nearly absent in Bale monkeys. The hybrid has an intermediate tail length and face colour, the face is darker in vervet but lighter in Bale monkey. However, this hybridization event is most likely as a consequence of the release of a pet vervet in the range of the FF population [J.-M. Lernould, personal communication]. (e, f) Adult vervet from Gonosa, Robe District with more golden fur, hands and feet are very dark, thick white browband, long and white whiskers, dark face with golden moustache, and long tail with black tip. (g, h) Adult Bale monkey in CF of Odobullu Forest exhibits a relatively brown/grey coat, lacks a white browband, whiskers are short, front neck covered with white fur, hands and feet blackish brown, and relatively short tail [12]. Bale monkey in FF of Kokosa (i) and Afura (j) that are phenotypically more similar to CF population (g, h) than that of grivet [12] and vervet (e, f). (DOCX 5055 kb) [file 12862_2018_1217_MOESM1_ESM.docx]

**
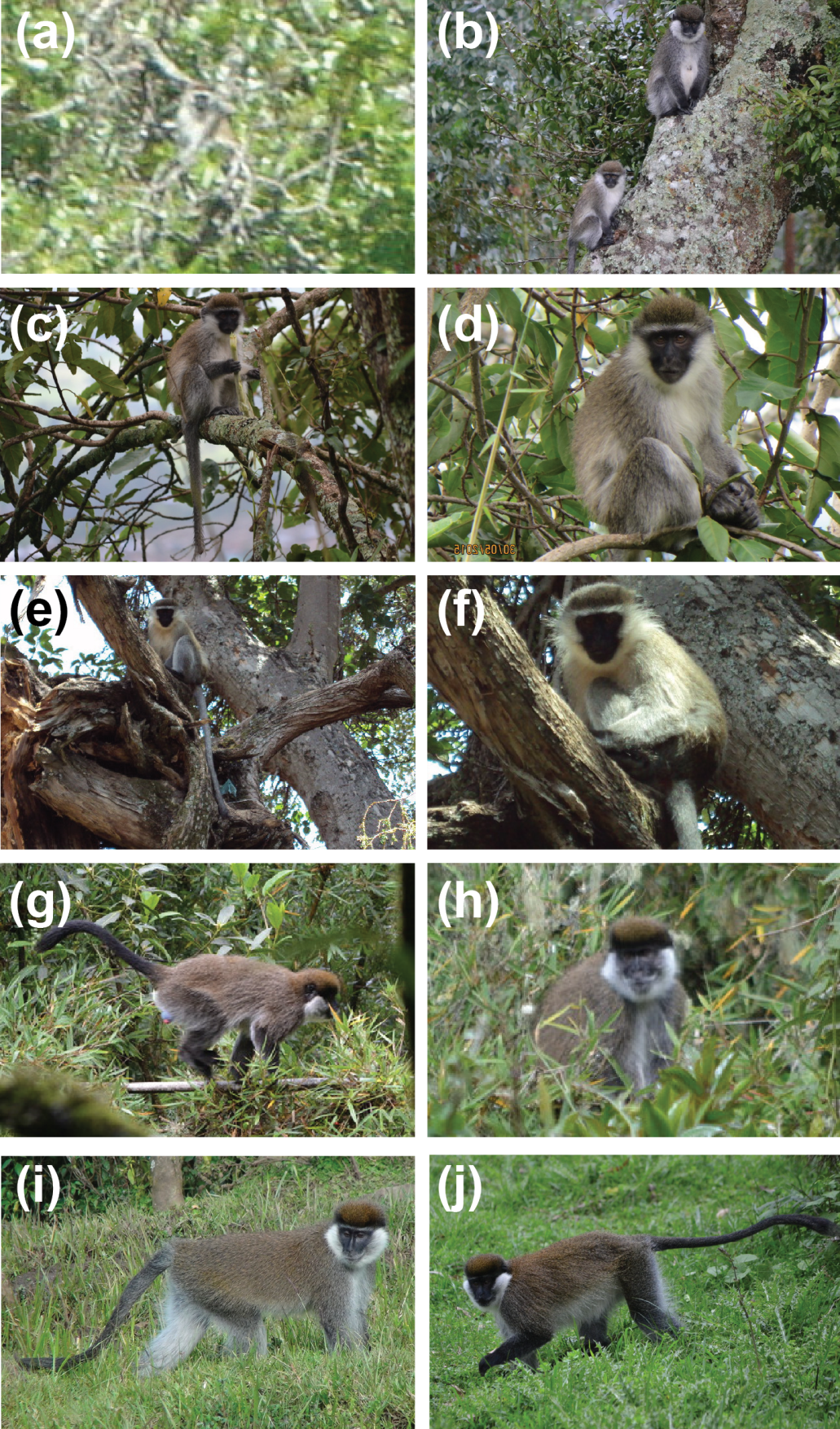
**

**Additional file 1** Comparison of the phenotypic appearance of pure Bale monkeys, vervets and their putative hybrids. (a) Adult putative vervet at Kokosa possibly the father of the hybrid. (b) Adult female Bale monkey and a putative juvenile hybrid of Bale monkey (♀) × vervet (♂) at Kokosa. A putative hybrid at Kokosa (c, d) with an intermediate coat colour that is more golden than that of a Bale monkey but less golden than that of a vervet. The animal shows a white browband, though it is smaller than that of a vervet and nearly absent in Bale monkeys. The hybrid has an intermediate tail length and face colour, the face is darker in vervet but lighter in Bale monkey. However, this hybridization event is most likely as a consequence of the release of a pet vervet in the range of the FF population [J.-M. Lernould, personal communication]. (e, f) Adult vervet from Gonosa, Robe District with more golden fur, hands and feet are very dark, thick white browband, long and white whiskers, dark face with golden moustache, and long tail with black tip. (g, h) Adult Bale monkey from Odobullu Forest exhibits a relatively brown/grey coat, lacks a white browband, whiskers are short, front neck covered with white fur, hands and feet blackish brown, and relatively short tail [[12](#_ENREF_12)]. Bale monkey in FF of Kokosa (i) and Afura (j) that are phenotypically more similar to CF population (g, h) than that of grivet [[12](#_ENREF_12)] and vervet (e, f).
