# Supplementary material for: Population genetic structure and evolutionary history of Bale monkeys (Chlorocebus djamdjamensis) in the southern Ethiopian Highlands
Source: BMC Evol Biol. 2018 Jul 10;18:106. doi: 10.1186/s12862-018-1217-y (PMC6038355; doi:10.1186/s12862-018-1217-y)
Supplement: Supplementary file 2 — Summary of sampling localities and number of samples. (DOCX 24 kb) [file 12862_2018_1217_MOESM2_ESM.docx]

**Additional file 2** Summary of sampling localities and number of samples

| No. | Sampling localities | Ccodes | Easting  Latitude | Northing  Longitude | Forest type | Taxon | Number of sequences | | Reference ID from [Haus et al. [20]](#_ENREF_20) |
| --- | --- | --- | --- | --- | --- | --- | --- | --- | --- |
|  |  |  |  |  |  |  |  |  |  |
| 1 | Odobullu | OD | 6.877215 | 40.175367 | CF | Bale monkey | 14 | |  |
| 2 | Shedem | SH | 6.812988 | 39.983206 | CF | Bale monkey | 10 | |  |
| 3 | Harenna | HR | 6.722921 | 39.741273 | CF | Bale monkey | 10 | |  |
| 4 | Afursa | AF | 6.707969 | 38.717650 | FF | Bale monkey | 11 | |  |
| 5 | Kokosa | KK | 6.733149 | 38.801435 | FF | Bale monkey | 14 | |  |
| 6 | Ekuma | EK | 6.793782 | 38.707582 | FF | Bale monkey | 11 | |  |
| 7 | Kulla | KL | 6.786365 | 38.679759 | FF | Bale monkey | 10 | |  |
| 8 | Gerbicho | GR | 6.577567 | 38.486055 | FF | Bale monkey | 10 | |  |
| 9 | Bokata | BO | 6.278838 | 38.649832 | FF | Bale monkey | 6 | |  |
| 10 | Gejaba | GJ | 6.241420 | 38.703291 | FF | Bale monkey | 10 | |  |
| 11 | Wotiye | WT | 6.278861 | 38.686147 | FF | Bale monkey | 3 | |  |
| 12 | Yeko | YK | 6.149111 | 38.733223 | FF | Bale monkey | 10 | |  |
|  | Total |  |  |  |  |  | **119** | |  |
|  |  |  |  |  |  |  |  |  |  |
| 1 | Lake Awassa | LA | 7.048088 | 38.462103 |  | Grivet | | 1 | aet (C2) 331 |
| 2 | Sodere | SD | 8.403206 | 39.392473 |  | Grivet | | 1 | aet (C2) 373 |
| 3 | Menagesha | MN | 8.965695 | 38.526452 |  | Grivet | | 1 | aet (C2) 485 |
| 4 | Woliso | WL | 8.535796 | 37.980229 |  | Grivet | | 2 | aet (C1) 463, 464 |
| 5 | Jimma | JM | 7.692432 | 36.806763 |  | Grivet | | 2 | aet (C2) 438, 439 |
| 6 | Bonga | BN | 7.266449 | 36.231309 |  | Grivet | | 1 | aet (C2) 451 |
| 7 | Arba Minch | AM | 6.912052 | 40.845828 |  | Vervet-grivet hybrid | | 2 | pyg x aet (C2) 391 - 87/391, 393 |
| 8 | Yabello | YB | 4.895958 | 38.070434 |  | Vervet | | 1 | pyg (C2) 421 |
| 9 | Sof Omar | SO | 6.038503 | 37.571891 |  | Vervet | | 1 | pyg (C6) 496 - 193 |
|  | Total |  |  |  |  |  | | **12** |  |

Sampling localities, coordinates and number of samples for (a) Bale monkey localities (this study) and (b) *Chlorocebus* samples collected by [Haus et al. [20]](#_ENREF_20) in the southern Ethiopian Highlands. Forest type: *CF* Continuous forest, *FF* Fragmented forest
