# Supplementary material for: Population genetic structure and evolutionary history of Bale monkeys (Chlorocebus djamdjamensis) in the southern Ethiopian Highlands
Source: BMC Evol Biol. 2018 Jul 10;18:106. doi: 10.1186/s12862-018-1217-y (PMC6038355; doi:10.1186/s12862-018-1217-y)
Supplement: Supplementary file 3 — Summary of haplotype distribution of Chlorocebus species. (DOCX 23 kb) [file 12862_2018_1217_MOESM3_ESM.docx]

**Additional file 3** Summary of haplotype distribution of *Chlorocebus* species

|  | Bale monkey - CF | | | Bale monkey - FF | | | | | | | | | aet | | | | | | pyg | | pyg x aet |  |
| --- | --- | --- | --- | --- | --- | --- | --- | --- | --- | --- | --- | --- | --- | --- | --- | --- | --- | --- | --- | --- | --- | --- |
| H | OD | SH | HR | KK | AF | EK | KL | BK | GJ | WT | YK | GR | LA | SD | MN | WL | JM | BN | SO | YB | AM | All |
| H1 | 10 |  |  |  |  |  |  |  |  |  |  |  |  |  |  |  |  |  |  |  |  | 10 |
| H2 | 4 |  |  |  |  |  |  |  |  |  |  |  |  |  |  |  |  |  |  |  |  | 4 |
| H3 |  | 2 |  |  |  |  |  |  |  |  |  |  |  |  |  |  |  |  |  |  |  | 2 |
| H4 |  | 5 |  |  |  |  |  |  |  |  |  |  |  |  |  |  |  |  |  |  |  | 5 |
| H5 |  | 3 |  |  |  |  |  |  |  |  |  |  |  |  |  |  |  |  |  |  |  | 3 |
| H6 |  |  | 10 |  |  |  |  |  |  |  |  |  |  |  |  |  |  |  |  |  |  | 10 |
| H7 |  |  |  |  |  | 1 | 2 |  |  |  |  |  |  |  |  |  |  |  |  |  |  | 3 |
| H8 |  |  |  |  |  |  | 3 |  |  |  |  |  |  |  |  |  |  |  |  |  |  | 3 |
| H9 |  |  |  |  |  |  |  |  |  |  |  | 2 |  |  |  |  |  |  |  |  |  | 2 |
| H10 |  |  |  |  |  |  | 1 |  |  |  |  |  |  |  |  |  |  |  |  |  |  | 1 |
| H11 |  |  |  |  |  |  |  |  |  |  |  | 5 |  |  |  |  |  |  |  |  |  | 5 |
| H12 |  |  |  |  |  |  |  |  |  |  |  | 1 |  |  |  |  |  |  |  |  |  | 1 |
| H13 |  |  |  |  |  | 10 | 4 |  |  |  |  |  |  |  |  |  |  |  |  |  |  | 14 |
| H14 |  |  |  |  |  |  |  |  |  |  |  | 2 |  |  |  |  |  |  |  |  |  | 2 |
| H15 |  |  |  |  |  |  |  | 6 | 4 | 3 | 10 |  |  |  |  |  |  |  |  |  |  | 23 |
| H16 |  |  |  | 14 | 11 |  |  |  | 6 |  |  |  |  |  |  |  |  |  |  |  |  | 31 |
| H17 |  |  |  |  |  |  |  |  |  |  |  |  | 1 |  |  |  |  |  |  |  |  | 1 |
| H18 |  |  |  |  |  |  |  |  |  |  |  |  |  | 1 |  |  |  |  |  |  |  | 1 |
| H19 |  |  |  |  |  |  |  |  |  |  |  |  |  |  | 1 |  |  |  |  |  |  | 1 |
| H20 |  |  |  |  |  |  |  |  |  |  |  |  |  |  |  | 2 |  |  |  |  |  | 2 |
| H21 |  |  |  |  |  |  |  |  |  |  |  |  |  |  |  |  | 2 |  |  |  |  | 2 |
| H22 |  |  |  |  |  |  |  |  |  |  |  |  |  |  |  |  |  | 1 |  |  |  | 1 |
| H23 |  |  |  |  |  |  |  |  |  |  |  |  |  |  |  |  |  |  | 1 |  |  | 1 |
| H24 |  |  |  |  |  |  |  |  |  |  |  |  |  |  |  |  |  |  |  | 1 |  | 1 |
| H25 |  |  |  |  |  |  |  |  |  |  |  |  |  |  |  |  |  |  |  |  | 1 | 1 |
| H26 |  |  |  |  |  |  |  |  |  |  |  |  |  |  |  |  |  |  |  |  | 1 | 1 |
| N | 14 | 10 | 10 | 14 | 11 | 11 | 10 | 6 | 10 | 3 | 10 | 10 | 1 | 1 | 1 | 2 | 2 | 1 | 1 | 1 | 2 | 131 |
| h | 2 | 3 | 1 | 1 | 1 | 2 | 4 | 1 | 2 | 1 | 1 | 4 | 1 | 1 | 1 | 1 | 1 | 1 | 1 | 1 | 2 | 26 |

Haplotype distributions within local populations of Bale monkeys, vervets, grivets and their hybrids in southern Ethiopia. H = Haplotype ID; N = Number of samples; h = number of haplotypes; aet = grivet; pyg = vervet; pyg x aet = vervet x grivet hybrid
