# Supplementary material for: Population genetic structure and evolutionary history of Bale monkeys (Chlorocebus djamdjamensis) in the southern Ethiopian Highlands
Source: BMC Evol Biol. 2018 Jul 10;18:106. doi: 10.1186/s12862-018-1217-y (PMC6038355; doi:10.1186/s12862-018-1217-y)
Supplement: Supplementary file 4 — FST values (p values) of pairwise comparisons among 12 Bale monkey localities. (DOCX 26 kb) [file 12862_2018_1217_MOESM4_ESM.docx]

**Additional file 4** F_ST_ values (p values) of pairwise comparisons among 12 Bale monkey localities

| Locality | Odobullu  (CF) | Shedem  (CF) | Harenna  (CF) | Yeko  (FF) | Gejaba  (FF) | Bokata  (FF) | Wotiye  (FF) | Afursa  (FF) | Kokosa  (FF) | Kullla  (FF) | Ekuma  (FF) | Gerbicho  (FF) |
| --- | --- | --- | --- | --- | --- | --- | --- | --- | --- | --- | --- | --- |
| Odobullu (CF) |  |  |  |  |  |  |  |  |  |  |  |  |
| Shedem (CF) | 0.624*** |  |  |  |  |  |  |  |  |  |  |  |
| Harenna (CF) | 0.995*** | 0.613*** |  |  |  |  |  |  |  |  |  |  |
| Yeko (FF) | 0.998*** | 0.914*** | 1.000*** |  |  |  |  |  |  |  |  |  |
| Gejaba (FF) | 0.996*** | 0.911*** | 0.998*** | 0.556** |  |  |  |  |  |  |  |  |
| Bokata (FF) | 0.997*** | 0.891*** | 1.000*** | **0.000** | 0.478* |  |  |  |  |  |  |  |
| Wotiye (FF) | 0.997** | 0.863** | 1.000** | **0.000** | **0.378** | **0.000** |  |  |  |  |  |  |
| Afursa (FF) | 0.998*** | 0.917*** | 1.000*** | 1.000*** | 0.350* | 1.000*** | 1.000** |  |  |  |  |  |
| Kokosa (FF) | 0.998*** | 0.928*** | 1.000*** | 1.000*** | 0.393* | 1.000*** | 1.000** | **0.000** |  |  |  |  |
| Kullla (FF) | 0.917*** | 0.826*** | 0.915*** | 0.523** | 0.515** | 0.444* | **0.340** | 0.542*** | 0.583*** |  |  |  |
| Ekuma (FF) | 0.968*** | 0.885*** | 0.969*** | 0.355*** | 0.268*** | 0.275** | **0.163** | 0.281*** | 0.321*** | 0.373* |  |  |
| Gerbicho (FF) | 0.934*** | 0.845*** | 0.933*** | 0.319*** | 0.328*** | 0.236* | **0.115** | 0.379*** | 0.423*** | 0.248* | 0.283** |  |

Significance pairwise differences: *** p < 0.001; ** p < 0.01; * p < 0.05 and bold represent no significant difference as in additional file 4.
